# Combination Therapy with Vitamin C Could Eradicate Cancer Stem Cells

**DOI:** 10.3390/biom10010079

**Published:** 2020-01-03

**Authors:** Noothan Jyothi Satheesh, Samson Mathews Samuel, Dietrich Büsselberg

**Affiliations:** Department of Physiology and Biophysics, Weill Cornell Medicine-Qatar, Education City, Qatar Foundation, Doha 24144, Qatar; noothanjyothi@yahoo.co.in (N.J.S.); sms2016@qatar-med.cornell.edu (S.M.S.)

**Keywords:** cancer stem cells, Vitamin C, ascorbic acid, cancer treatment, combination therapy, reactive oxygen species

## Abstract

Cancer remains one of the most feared and dreaded diseases in this era of modern medicine, claiming the lives of many, and affecting the quality of life of several others around the globe despite major advances in the diagnosis, treatment, palliative care and the immense resources invested into cancer research. While research in cancer has largely focused on the neoplasm/tumor and the cancerous cells that make up the tumor, more recently, the existence, proliferation, differentiation, migration and invasion of cancer stem cells (CSCs) and the role that CSCs play in tumor initiation, progression, metastasis, drug resistance and relapse/recurrence of the disease has gained widespread interest in cancer research. Although the conventional therapeutic approaches such as surgery, chemotherapy and radiation therapy are effective cancer treatments, very often these treatment modalities fail to target the CSCs, which then later become the source of disease recurrence. A majority of the anti-cancer agents target rapidly dividing cancer cells and normal cells and hence, have side effects that are not expected. Targeting CSCs remains a challenge due to their deviant nature with a low proliferation rate and increased drug resistance mechanism. Ascorbic acid/Vitamin C (Vit.C), a potent antioxidant, is a cofactor for several biosynthetic and gene regulatory enzymes and a vital contributor to immune defense of the body, and was found to be deficient in patients with advanced stages of cancer. Vit.C has gained importance in the treatment of cancer due to its ability to modulate the redox status of the cell and influence epigenetic modifications and significant roles in HIF1α signaling. Studies have reported that intravenous administration of Vit.C at pharmacological doses selectively kills tumor cells and targets CSCs when administered along with chemotherapeutic drugs. In the current article, we provide an in-depth review of how Vit.C plays an important role in targeting CSCs and its possible use as an adjuvant, neoadjuvant or co-treatment in the treatment of cancers.

## 1. Introduction

According to World Health Organization (WHO) global statistics, cancer is the second main cause of death [1]. In 2018, it was estimated that around 9.6 million people worldwide died due to cancer. The most prevalent types of cancer in women include breast, colorectal, lung, cervical and thyroid cancer, while for men it includes lung, prostate, colorectal, stomach and liver cancer. Although great progress has been made in understanding the underlying pathophysiology of cancer, cancer detection and treatment strategies, a proper cure regime has still not yet been revealed [2]. Current treatment regimens result only in limited survival rates for most advanced stage cancers, as these treatments mainly target the tumor load and not cancer stem cells (CSCs) [3,4].CSCs are a smaller population of cells present in tumor loads of different types of cancers [5] like breast cancer, brain tumors, colorectal cancer, prostate cancer, lung cancer, and melanoma [6]. CSCs represent a minor subpopulation of about 0.001–0.1% of the whole tumor mass, but are considered as the key factor for cancer recurrence [6]. They are responsible for cancer reoccurrences as they reside in the tumor load [5]. They constantly adapt their energy metabolism to the surrounding micro-environmental change by expediently shifting their energy source or production from one pathway to another, or by attaining an intermediary metabolic phenotype [7].

**Figure 1:**
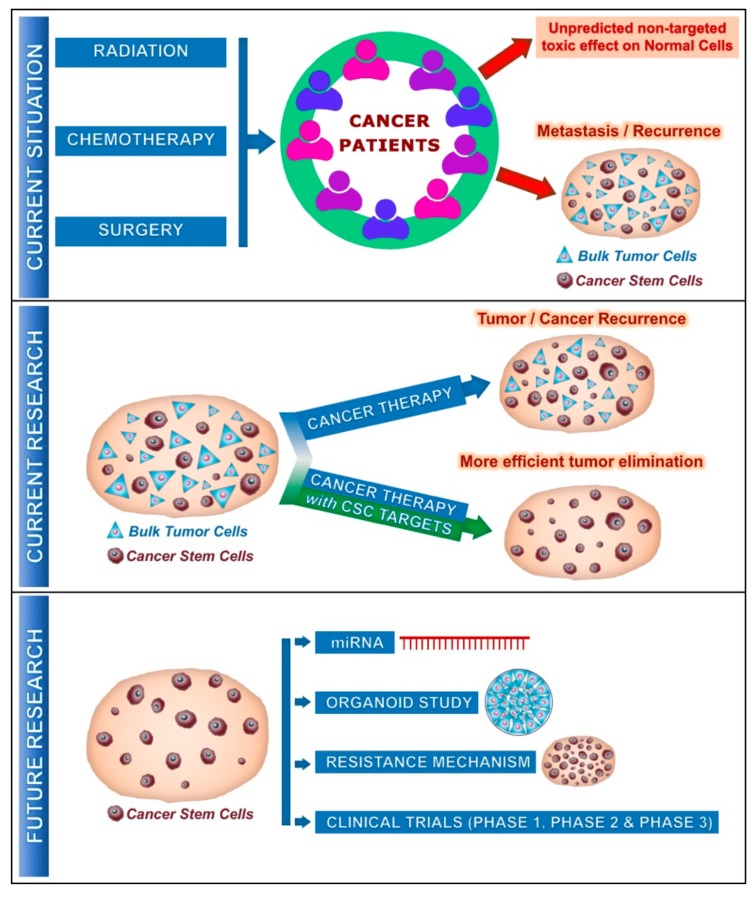
Current and future involvement of cancer stem cells (CSCs) on cancer treatment: Current situation describes the effect of cancer therapy on unpredicted non-targeted effects on normal cells and metastasis/recurrence of cancer after several years due to the presence of CSCs along with tumor cells. Current research reveals that standard cancer therapy with CSC targets provides much more efficient outcomes on the tumor progression with elimination of CSCs. In the future, further studies could be focused on miRNA (microRNA), cancer organoid, resistance mechanism by CSCs and could enter the clinical phases, promising a better outcome for the cancer patients.

Although, it’s known that the usual cancer therapies target the largely fast-growing neoplastic cells, failure in cancer therapies often urges the need to understand the debatable role of CSCs. It’s understood that CSCs exist during cancer progression and metastasis and that they have low proliferation rate and high drug resistance [2,8], which makes them easier to escape the existing cancer treatments. All conventional cancer therapies like hormonal therapy, surgery, immunotherapy and anti-angiogenesis therapy do not succeed in respect of the long-term effect for mainly two reasons. (1) All these treatments fail to target the CSCs, and (2) due to the unpredictable non-targeted toxic effect on the normal cells [2] (Figure 1). Recent studies have shown that intravenous administration of Vitamin C (Vit.C), along with the conventional cancer therapy, is successful in decreasing cancer progression and is a great hope for many cancer patients around the world [9]. This review briefs on the effect of Vit.C on cancer cells and their potential effect on CSCs.

## 2. Cancer Stem Cell Metabolism

Similar to normal stem cells, CSCs exhibit characteristics like self-renewal, expression of markers, cluster of differentiation (CD), surface markers like CD24, CD133, CD44, CD49, CXCR4 (C-X-C chemokine receptor type 4) and Leucine-rich repeat-containing G-protein coupled receptor 5 (LGR5) or intracellular markers like aldehyde dehydrogenase (ALDH) [6,10]. Table 1 shows the CSC markers present in various types of cancer. Major signaling pathways activated in CSCs include Wnt, JAK/STAT, Notch, PI3K/AKT and Hedgehog signaling [6,10]. CSCs are known to be in the resting G0 phase of the cell cycle and are in a quiescent state, and hence escape the cancer treatment that mainly targets the highly proliferating cancer cells [6,11]. Hence, cancer therapy must also target these potent small populations of CSCs for better treatment regimes. Identical to cancer cells, CSCs also undergo metabolic alterations. CSCs are managed by bioenergetic signaling pathways such as fatty acid metabolism, glutamine metabolism and the AKT-mTOR pathway. They undergo metabolic alterations in lipid metabolism, glycolytic activity and mitochondrial respiration. In addition, hypoxia—the crucial factor in malignancy, chemo resistance and poor survival rate of cancer patients—leads to the maintenance of an undifferentiated state that affects the proliferation and fate of the normal stem cells [6]. This opens the scope for focusing on CSC metabolism strategy for self-renewal, quiescence and cell division to develop a more robust and effectual cancer therapy with lesser chances of metastasis and recurrence [6,11,12].

Studies have also shown that non-coding RNAs like miRNAs (21–25 nucleotide long non-coding RNAs that regulate the gene expression at the post-transcriptional levels) play a role in various cancer types and are involved in the regulation of CSCs, which includes properties such as CSC cell division, tumorigenesis and CSC drug resistance [13,14]. Various types of miRNA that are present in CSCs are briefed in Table 1.

## 3. Vitamin C/Ascorbic Acid—Metabolism and Chemistry of Vitamin C

Vit.C, also known as ascorbic acid, is a water-soluble vitamin present naturally in many foods. All plants and most animals synthesize Vit.C, whilst for humans and some primates, it needs to be supplemented via diet [76]. A nutritional site (https://www.healthline.com/nutrition/vitamin-c-foods#section21) claims 90 mg to be the daily value of Vit.C required for humans. Some of the most common, rich sources of Vit.C include green chili peppers (242 mg/100 g), guavas (228 mg/100 g), sweet yellow papers (183 mg/100 g), black current (181 mg/100 g), thyme (160 mg/100 g), parsley (133 mg/100 g), kiwis (93 mg/100 g) and lemon (77 mg/100 g).

In 1928, Albert Szent-Gyorgyi was one of the first people who first isolated Vit.C, while in 1932, Szent-Gyorgyi and King discovered its antiscorbutic factor [76,77,78]. Vit.C deficiency could lead to a fatal disease called Scurvy if left untreated, and could only be cured by Vit.C administration [76].

Vit.C is a potent electron donor (reducing agent) and these released electrons play the central role in its physiological effects [76]. As these electrons (two electrons) released from Vit.C can reduce oxidants/oxidized species, they are categorized as an antioxidant, but with a twist. Vit.C released electrons could reduce metals such as iron and copper, and release superoxide and hydrogen peroxide which in turn leads to the production of reactive oxidants. Thus, in certain conditions, the end product of Vit.C action as a reducing agent would produce oxidants [76]. This was observed in both in vitro conditions, when physiological concentration of Vit.C is present along with the metals, and in vivo conditions, when pharmacological concentration (millimolar range) of Vit.C are achieved in extracellular fluids and in plasma [76,79].

Under physiological conditions, Vit.C exists in the state as ascorbate anion and donates two electrons from the double bonds at carbon two and three. Oxidation of ascorbate anion through the loss of the first electron releases the free radical-ascorbate radical/semi-dehydroascorbic acid, and is a reversible process. In animals, Vit.C catabolism products further enter the pentose phosphate pathway or other pathways of carbohydrate metabolism [19], hydrogen peroxide can be formed, while ascorbic acid is found in millimolar concentrations, together with the presence of metal ions.

The putative enzymatic role of Vit.C causes it to act as a cofactor to several enzymatic reactions [80]. It acts as an electron donor in numerous enzymatic reactions such as peptide hormone amidation, collagen hydroxylation (adding hydroxyl groups to proline or lysine residues to strengthen the collagen triple helix) and norepinephrine synthesis. During this enzymatic reaction, ascorbic acid retains the prosthetic metal ions of these enzymes (i.e., ferrous ion (Fe^2+^) and cupric ion (Cu^2+^) in their reduced forms [80,81].

## 4. Transporters of Vit.C

In vivo, Vit.C transport occurs via special transporters called sodium dependent Vit.C transporter (SVCT) 1 and 2 [76,82,83,84,85]. They belong to a nucleobase transporter family and are highly conserved. Studies on the mRNA expression of these transporter proteins revealed the distribution of them in humans and animals [76]. SVCT1 and SVCT2 transport ascorbic acid, but not dehydroascorbic acid (DHA) into the cells. Transportation of DHA to the cells is mainly done by glucose transporters—GLUTs (GLUTs 1–4 and 8) [76,86,87,88,89]. Certain GLUTs show a higher affinity to DHAs over glucose [76]. However, the transporter that transports Vit.C byproducts from cells to the extracellular fluid or plasma is still not understood. Mature red blood cells are the only ascorbate containing cells that lack SVCT transporters and this area needs to be further investigated. Red blood cells attain their ascorbate by the transportation of DHA and are internally immediately reduced. In humans, GLUT1 transports DHA into the red blood cells, while in mice, DHA is transported by GLUT 3 and/or 4 [76,90]. SVCT1 is mainly expressed in absorptive tissues like intestinal epithelium, in kidneys—at the proximal convoluted tubules and the descending part of the loop of Henle. SVCT1 is also present in skin, the liver and the lungs. However, SVCT2 and GLUTs are present in most body tissues, including the brain, pituitary gland, thyroid, heart, adrenals, skeletal muscles, spleen, stomach, pancreas, bladder, ovaries and testis in addition to the tissues where SVCT1 is present.

## 5. Evidence for Vit.C Effect in Cancer Treatment

In the general population, Vit.C deficiency is a rare condition, however its commonly observed in advanced cancer patients [91,92] This may be due to the insufficient oral intake of Vit.C, lower bioavailability, increased tissue utilization and increased oxidative stress [93].Vit.C is known for its vital role for many elemental biochemical processes, and as a source for reduced iron which is an imperative factor for proper functioning of the epigenetic regulators which in turn instigate the DNA and histones demethylation [91]. Epigenetic modification is a crucial mediator in cancer as it triggers and maintains the malignant phenotype characteristics of cancer [91,94]. Several invitro studies confirmed that physiological concentrations of Vit.C along with a hypomethylation compound would have a synergistic effect directly or indirectly on DNA demethylation [91]. In addition, recent studies confirmed that the intravenous administration of Vit.C at pharmacological doses selectively kills tumor cells [91,93].

In the human body, apart from the individual’s health status, bioavailability of Vit.C is controlled by intestinal and renal absorption, tissue stores and renal excretion. Levels of Vit.C range between 300 mg–2 g in the human body, with 300 mg during conditions such as scurvy. The normal range for ascorbate in human blood plasma is 0.70–1.4 mg/dL, while oral intake of Vit.C generates maximum serum levels of 1.3–4.0 mg/dL (73.8–227.1 μmol/L) whilst the intravenous administration of Vit.C maximizes the levels to 350 mg/dL (19.873 mm mol/L). The unit conversion for Vit.C levels is 1 mg/dL = 56.78 μmol/L [9]. Studies have confirmed that the high plasma concentrations of Vit.C could only be achieved via intravenous administration as the intestinal absorption of Vit.C is limited with oral intake. Vit.C exerts its antioxidant and pro-oxidant activity at low and high concentrations, respectively [95]. Vit.C acts as an electron donor and protects the body from oxidative stress via different routes. It protects plasma lipids from peroxyl radicals and protects the body against aqueous radicals that are present in the blood [95,96]. Vit.C exerts its pro-oxidant properties via intracellular reactive oxygen species (ROS) levels, induction of endoplasmic stress, inhibiting and suppressing the production of the angiogenic factor and insulin-like growth factors, respectively [97].

Recent research trends in cancer therapy have focused on in vitro and in vivo studies where the effect of high-dose Vit.C alone or in combination with radiation, chemotherapy (paclitaxel, cisplatin, carboplatin, azacitidine) or other drugs (doxycycline, azithromycin) is researched. The study details are briefed in Table 1.

## 6. Vit.C and Its Anticancer Mechanism

Several studies during the past decade confirmed that pharmacological concentrations of Vit.C in the millimolar range are effective in in vitro studies as they kill cancer cells, and in vivo by slowing down the tumor growth [9,98]. However, the mechanism behind the sensitivity of cancer cells to Vit.C and resistance of normal cells to Vit.C is still not clearly understood and needs further investigation. Since Vit.C controls various processes, the activity of Vit.C is also dependent on several different factors like cancer type and signaling pathways included in the tumor development. Ngo et al. proposed three different characteristics in cancer that the pharmacological levels of Vit.C could target to exert its effect on cancer metabolism [99]. These include Vit.C targeting the redox imbalance, epigenetic regulators and HIF1 signaling.

### 6.1. Redox Imbalance

In cancer cells, due to defective mitochondria and increased metabolic rate, oxidative stress is more in comparison to the normal cells. [99,100]. As known, ROS could increase the imbalance in the genetic make-up that facilitates tumor growth and also increase cell proliferation. Increased levels of ROS could be a danger to the same cancer cells. To escape this effect of ROS, and to compensate the ROS effect, cancer cells increase or improve other signaling pathways [99,101]. Hence, as ROS encourages the development of cancer, antioxidant treatments have to be considered as an anticancer treatment strategy, and more studies are needed to confirm the antioxidant induced cancer suppression. Conversely, a reverse effect of antioxidant therapy has been observed in both in vivo and clinical studies, with an increase in cancer growth in mouse models of melanoma and lung adenocarcinoma, and in lung and prostate cancer patients, respectively. [99,102,103]. Hence, it is assumed that certain types of cancer would be favored by antioxidants and would be susceptible to pro-oxidant therapy. Pro-oxidants induce oxidative stress either by ROS production or by inhibiting the antioxidant systems [99,104], and include therapies like radiation for pro-oxidant anticancer therapy. Despite of all these improvements in cancer treatments, the pro-oxidant therapy has also not been found to be completely effective and could lead to reduced effects of therapeutics [99,105]. Vit.C could potentially evade this issue due to two common characteristic features of cancer cells, which include the increased levels of labile metals like iron [99,106] and increased uptake of glucose and glycolysis (DHA via GLUT1) [99,107]. These two mechanisms could occur simultaneously in cancer cells, thereby inducing a synergistic effect on Vit.C on cancer cells.

#### 6.1.1. Increased Levels of Labile Metals Like Iron

Vit.C induce its pro-oxidant activity in the vicinity of redox-active metals like iron. In cytosol and mitochondria, the labile iron as ferrous iron (Fe^2+^) (reduced state) (1 µM in humans), reacts with H_2_O_2_ (Hydrogen peroxide) releasing hydroxyl radical (OH) by Fenton reaction [99]. Vit.C helps in this reaction by donating electrons to the oxidized state of iron-ferric iron (Fe^3+^) to generate Fe^2+^, resulting in ROS production and hence cell death. Iron containing enzymes and haem containing enzymes play a vital role in DNA synthesis, epigenetics, cell cycles and cellular respiration, and hence cancer cells highly depend on labile Fe^2+^ state iron for their growth and survival. Breast cancer cells have almost twice the storage capacity of intracellular labile Fe^2+^ state iron than normal breast epithelial cells, and this is also similar in prostate and lymphoma [99,108,109]. Thus it is evident that compared to the normal cells, as cancer cells produce more H_2_O_2_ and OH, they become more susceptible to Vit.C [99].

#### 6.1.2. DHA Uptake Instead of Glucose

**Figure 2:**
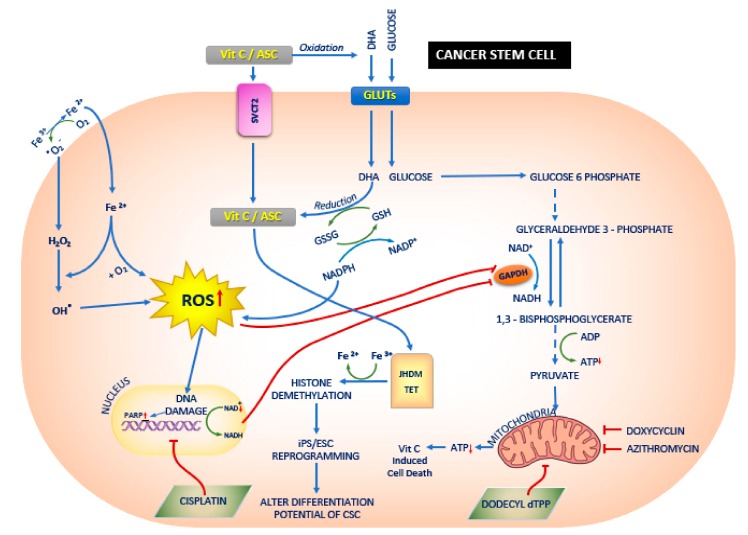
The effect of Vitamin C (Vit.C) on cancer stem cells (CSCs). Vit.C along with the conventional cancer therapy has a synergistic effect on the treatment of cancers. Vit.C enters the CSC via sodium dependent Vit.C transporter 2 (SVCT2) or glucose transporters GLUTs, and thereby alters jumonji-C domain-containing histone demethylases (JHDM)/ Ten eleven translocation (TET) and reactive oxygen species (ROS) respectively. This leads to mitochondrial dysfunction and also alters the differentiation potential of the CSCs. In CSCs [117], glucose enters the cells via GLUTs [111] and a series of downstream processes occurs: ROS generation is increased via regulation of Glutathione (GSH) and Nicotinamide adenine dinucleotide phosphate (NADPH); DNA damage and increase in poly (ADP-ribose) polymerase (PARP) occurs; both increase in ROS and increase in PARP leads to the inhibition of glyceraldehyde 3-phosphate dehydrogenase (GAPDH) [111]. Vit.C enters the CSCs via SVCT2 [98] and increases JHDM and TET [118], leading to histone demethylation [119] and Induced pluripotent stem cells (iPS)/ embryonic stem cells (ESCs) reprogramming [118]. In addition, Ferrous (Fe3+) ion reduces to ferric (Fe^2+^) ion and enters the CSCs as Fe^2+^ and hydrogen peroxide (H2O2), thereby increasing the ROS production. Studies have shown the doxycyclin [117,120], azithromycin [20] and dodecyl tri-phenyl-phosphonium (dTPP) [121] inhibit mitochondrial activity which in turn leads to Vit.C induced cell death.

The Warburg effect that explained the dependency of cancer cells to glucose and glycolysis was described as a fundamental mechanism for the proliferation and survival of cancer cells [99,107,110]. Mutations of proto-oncogenic genes—KRAS (Ki-ras2 Kirsten rat sarcoma viral oncogene homolog, encoding a small GTPase superfamily protein) and BRAF (encoding a protein belonging to the RAF family of serine/threonine protein kinases which plays a key role in regulating the MAP kinase/ERK signaling pathway) also induce the Warburg effect along with GLUT1 up regulation [99,111]. A recent study in KRAS and BRAF mutant colorectal cancer cells, showed the effect of a high dose of Vit.C on exploiting the vulnerability of cancer cells on glucose and glycolysis. Due to the structural similarity of DHA—an oxidized form of Vit.C/ascorbic acid and glucose—they are taken up by KRAS and BRAF mutant cells, and the DHA is then reduced back to ascorbic acid by glutathione (GSH) and nicotinamide adenine dinucleotide phosphate (NADPH) in the intracellular environment, which leads to a decrease in intracellular antioxidants and increased ROS levels. The increased levels of ROS deactivate glyceraldehyde 3-phosphate dehydrogenase (GAPDH), a glycolytic enzyme, and activate poly (ADP-ribose) polymerase (PARP) [99,112], which in turn leads to the reduction of NAD+ (Nicotinamide adenine dinucleotide), a cofactor of GAPDH, and hence inhibits GAPDH further [99,113] (Figure 2). Thus, the inhibition of GAPDH in highly glycolysis dependent KRAS or BRAF mutant cells leads to a state of energy crisis and hence results in cell death, when compared to wild type cells.

### 6.2. Ten Eleven Translocation (TET)—Cancer and Effect of Vit.C (Targeting Epigenetic Regulators)

Ten eleven translocation (TET) proteins are 180–230 kDa large multidomain enzymes. All TET proteins are conserved with a cysteine-rich domain, double-stranded β-helix (DSBH) domain and cofactor binding sites for Fe(II) and 2-oxoglutarate (2-OG) which together form the main catalytic domain in the C-terminus [114]. Structural studies confirmed that these catalytic domains specifically bind to cytosines in CG dinucleotides in DNA, known as CpG sites and do not bind to other DNA bases and have almost no specificity to the flanking DNA sequences [114]. The three family members of the TET family—TET1, TET2 and TE3—catalyze the successive hydroxylation of DNA methyl cytosine 5-methylcytosine (5-mC) to 5-hydroxymethylcytosine (5-hmC), 5-formylcytosine (5-fC) and 5-carboxycytosine (5-caC) [115]. These 5-mC oxidation products 5-fC- and 5-caC-modified cytosine are the intermediates that are formed during the conversion of 5-mC to unmodified cytosine, impending for the first step for active DNA demethylation pathway [114,115]. De novo 5′methylation of cytosine is induced by DNA methyltransferase 3A (DNMT3A) and DNMT3B: 5-mC is maintained by methyltransferase DNMT1 [116]. 

The dysregulation of epigenetics, including DNA methylation, is a characteristic feature of cancer [114,122]. Recent studies have confirmed that in most cancers, DNA methyl cytosine (5-mC) are highly disturbed [122,123,124,125]. Several studies focused on the roles of the epigenetic regulators like DNMTs, TETs and isocitrate dehydrogenase (IDH) enzymes in gene expression, development, cellular development and transformation [122,126] and interestingly TET enzymes were noticed as a vital tumor suppressor mechanism in cancer [114]. TET2 is categorized as one of the most frequently categorized malignancies, and TET2 alteration is considered as an early onset of cancers [114]. The studies show that in various cancer types, mutation of all three TET genes, with reduced expression and impaired activity of the proteins, are present. Thus, it is concluded that defined regulation of DNA methylation patterns are partially regulated by TET enzymes which are vital for the normal development and fundamental protection against cellular transformation [114].

Enzymatic activity of TET enzymes is increased by Vit.C. Several in vitro studies confirmed the effect of Vit.C on increasing the DNA methylation in a TET dependence manner [116,127,128]. The stimulatory effect of Vit.C of TET activity is proposed by two mechanisms. The first mechanism proposes the role of Vit.C as an enzyme cofactor that directly binds to the catalytic domain of the TET enzyme which would increase enzymatic activity of these enzymes along with promoting the TET folding to improve the Fe(II) recycling [129]. In addition, in vitro studies also found that only Vit.C, and no other antioxidants, shows an effect of TET enzyme activity [128,129,130]. A second mechanism proposes the role of Vit.C as a stimulant on the TET enzymatic activity and is associated with the ability of Vit.C to promote reduction of Fe^3+^ to Fe^2+^ [119]. This mechanism is supported by similar effects, by compounds like redox-active quinone stimulating TET activity, by reducing iron-free Fe^3+^ to Fe^2+^ [119].

In human melanoma cell lines, physiological Vit.C concentration of 100 μM increased the concentration of 5-hmC to comparable levels of normal melanocytes and further reduced the malignant effect of these cells, without interfering the proliferation of these cells [116,131]. These low and physiological Vit.C concentrations of 100–200 μM induce apoptosis in certain melanoma cells via epigenetic downregulation of clusterin, leading to Bax activation and Bcl-XL sequestering in the mitochondria, which in turn result in apoptosis [116,132].

### 6.3. Hypoxia-Inducible Factor (HIF)—Cancer and Effect of Vit.C

Hypoxic conditions are often observed in solid tumors [116]. Hypoxia-inducible factor (HIF) is a heterodimeric transcription factor that hallmarks many cancers [133]. HIF intervene on several cancer progression processes, like epithelial-mesenchymal transition (EMT), angiogenesis, maintenance of stem cells, invasion and metastasis of cancer cells, resistance of cancer cells to chemotherapy and radiation therapy [116,134].

Under normal oxygen levels, Vit.C down regulates the HIF-1α unit via a Vit.C dependent hydroxylases, while on the other hand, under hypoxic conditions this process is reversed. Under hypoxic conditions, suppression of HIF-1α, hydroxylation is observed which leads to an amplification in HIF dependent gene transcription, neo-angiogenesis, tumor development and progression [116,135]. However, it’s interesting to notice that lower levels of Vit.C advance tumor growth and progression by reducing HIF-1α hydroxylation, thereby stabilizing the HIF-1α levels. Moreover, higher levels of HIF make cancer cells more sensitive to toxicity induced by Vit.C [116,135].

## 7. Synergetic Effect of Vit.C on Energy Metabolism in Cancer Stem Cells

A possible effect of Vit.C on CSCs propagation has drawn attention due to two mechanism of action of Vit.C in cancer cells [117] (Figure 2). Firstly, due to its potent pro-oxidant role that depletes the glutathione levels causing cellular oxidative stress and apoptosis, and secondly, due to its inhibitory role on glycolysis via targeting GAPDH, the key enzyme in the glycolytic pathway. These two mechanisms only gives us little knowledge in understanding the effect of Vit.C on CSC propagation, and hence, it is essential that new doors are unlocked and further research in this area is undertaken [117]. 3D mammosphere formation of 3 spheroid culture in breast cancer cell line MCF7 confirmed that a glycolysis inhibitor like Vit.C reduces stemness with an IC-50 of 1 mM [117]. This study also showed the NADH auto-fluorescence as a new biomarker for identifying CSCs [117]. Furthermore, the studies demonstrated the synergistic effect of Doxycycline and Vit.C as an effective combination therapy for eradicating CSCs, as Doxycycline inhibits the mitochondrial biogenesis and OXPHOS while Vit.C inhibits the glycolysis pathway via inhibiting GAPDH. Thus a combination therapy has a much greater effect in CSC eradication [120]. 

A triple combination therapy using lower doses of two clinically approved drugs—Doxycycline and Azithromycin—at a concentration of 1 μM and a dosage of Vit.C of 250 μM, showed a complete eradication of CSC propagation [20] (Figure 2). The synergistic effect of these compounds on the breast cancer cell line MCF7 were induced by the inhibition of two key targets, namely the large (39 s) and small mitochondrial ribosomes (28 s). Although Vit.C generally acts as an antioxidant, due to its relative concentration and cellular localization, they acted as a mild pro-oxidant and stimulated free radical production leading to mitochondrial biogenesis in addition to mitochondrial oxidative stress. However, in this study no Vit.C inhibiting glycolysis via inhibiting glycolytic enzyme GAPDH was observed [20].

A recently published article reemphasized the effect of Vit.C on eradicating CSCs. The effect of the integrated metabolic strategy to exterminate CSCs was validated [121]. Breast cancer cells MCF-7 and MDA- MB-231 were treated with mitochondria—a targeted small non-toxic compound Tri-Phenyl-Phosphonium (TPP) derivative called Dodecyl—TPP (d-TPP) (Figure 2). This TPP derivative is a powerful compound to block the mitochondrial function and reduces the cells viability of CSCs in a dose and time-dependent manner. The study also proves that d-TPP inhibits the formation of 3D mammosphere formation, a read-out for the CSC activity and proliferation. Metabolic flux analysis using Seahorse analyzer confirmed the inhibitory effect of d-TPP on mitochondrial basal respiration and ATP production and exhibited a transition to the glycolytic pathway to complement the effect on mitochondria. However, it was also observed that not only the d-TPP switches the energy pathway in these breast cancer cell lines, but also the sensitivity to other metabolic inhibitors like Vit.C and 2-DG (glycolysis inhibitors) and Doxycycline, Niclosamide, and Berberine (OXPHOS inhibitors). Thus this study conferred a synergistic effect, a “Two-Hit” approach by metabolic inhibitors on the CSC propagation, and has proven that Vit.C is a potential compound to target the glycolysis pathway for this effect on CSCs [121].

In vivo assay confers the effect of Vit.C on hepatocellular carcinoma (HCC) by showing a reduced effect on the self-renewal capacity, survival, CSC associated gene expression on CSC upon Vit.C treatment [98]. They showed the effect of Vit.C on HCC cells via DNA damage and depletion of ATP leading to the activation of cyclin-dependent kinase inhibitor p21 which in turn leads to the G2/M phase cell cycle arrest and caspase-dependent apoptosis. The study also focused on the synergistic effect on killing HCC cells of Vit.C along with cisplatin, a chemotherapeutic drug on both in vitro and in vivo conditions. Despite DNA damage caused by Vit.C induced ROS, cisplatin induced DNA damage via the reaction of platinum molecule at the nucleophilic sites and hence combined effect of both cisplatin and Vit.C had an extended effect on inducing DNA damage in HCC than single effect [98] (Figure 2). Furthermore, it was confirmed that in liver CSCs, SVCT-2 is highly expressed and could be used as a biological marker for the liver CSCs and the effect of Vit.C would be via SVCT-2 [98].

## 8. Role of Vit.C in Cancer Epigenome Regulation

DNA methylation, covalent addition of methyl group at cytosine within CpG dinucleotides at CpG islands is associated with regulation of gene expression and gene silencing to regulate the genome functioning by histone modification. It is understood that hypermethylation at the promoter regions off certain tumor-suppressor genes leads to its inactivation and gene silencing in different types of cancer [118]. In cancer cells, reinstating the TET function by Vit.C, in combination with certain epigenetic targeted therapies and hypomethylation compounds would help to expunge the epigenetic memory of the cancer cell state. Hence, the epigenome for these cells would resume its normal potential to differentiation and expression of tumor suppression gene [136].

The biological network of cell development is altered by Vit.C by reprogramming epigenome commitment in a reverse effect, by inhibiting senescence and by maintaining the differential potential. Cells epigenome is hypermethylated during normal growth environment with low levels of Vit.C, as the activity of a-ketoglutarate dependent dioxygenases (a-KGDDs) including Jumonji-C domain-containing histone demethylases (JHDMs) and TET proteins are inhibited (Figure 2). With an increase in Vit.C concentration, the enzymatic activity of JHDM and TET is increased, and this in-turn leads to the loss of histone and DNA methylation. This could facilitate the differentiation of embryonic stem cells (ESCs) to a blastocyst-like ESC state, somatic cell reprogramming, differentiation or cancer cell death and guard the adult cells from senescence and aging. Hence, it is concluded that Vit.C manipulates the epigenetic memory and alter the differentiation potential of cancer cells and cancer stem cells.

## 9. Future Studies on Vit.C on Cancer Stem Cells

Effective combination treatment strategy with Vit.C and chemotherapeutic drug is currently showing a good perspective in treating various cancers and hence the survival time of cancer affected patients could get better. However, this effect needs to be further investigated as only a little is being studied so far (Table 1). Certain miRNAs and their role in CSCs function is understood in several cancers like breast cancer, prostate cancer, osteosarcoma, liver cancer etc. (Table 1) [18,22]. Further research should focus on the effect of Vit.C on miRNAs associated with CSCs. In addition, organoid study on the CSCs isolated from peripheral blood mononuclear cells (PBMCs) [137] could be used to understand the effect of Vit.C on cancer stem cell progression in several cancer types and could give much better knowledge on the same (Figure 1). In addition, research should also focus on the possible resistance mechanism that CSC could develop to escape the Vit.C combination therapy (Figure 1).

## 10. Conclusions

Relapse of cancer on cancer patients is a challenge in cancer treatment and makes the cancer medical care challenging and greatly expensive. Researchers observed that conventional therapies fail in cancer treatment when they do not target CSC. Moreover, normal cells show toxic effects. Administration of Vit.C along with other chemotherapeutic drug proves to be an additional and effective treatment mechanism. Vit.C induces its effect via interfering the energy metabolism or cancer epigenome regulation in cancer stem cells. Several studies confirmed that combination therapy of Vit.C along with conventional therapy has a greater impact on cancer growth or progression and should be considered as a future treatment strategy. Further studies on their effect of miRNAs, organoid cultures and possible resistance mechanism will help to better understand the treatment strategies of combination therapy using Vit.C for eradiating cancer stem cell progression in various types of cancers. This will not only save money, but will also reduce the suffering of cancer patients and their families.

## Figures and Tables

**Table 1:** Current research status on the effect of Vit.C on various cancer types. It also represents the various cancer stem cells (CSC) markers and miRNA associated with various cancer types.

Type	In Vitro/In Vivo	Status/Month Year	Drugs	Reference	CSC Markers	Reference	miRNA in CSC	Reference
**Breast Cancer**	In vivo, Population based cohort	Completed/April 2006	Chemotherapy, Radiation, Vit.C/E, Multivitamin	[15]	ALDH1	[2,16,17]	miR-495	[18,19]
	In vitro, MCF-7 cells	Published/2019	Doxycycline, Azithromycin, Vit.C	[20]	CD44	[2,16,21]	miR-7	[22,23]
					CD24	[16]	miR-34a	[22,24]
					CD133	[16]	miR-181	[18,25]
					CD90	[16]		
					α6-integrin	[16]		
					Hedgehog-Gli activity	[16]		
**Pancreatic Cancer**	In vivo/Phase 2 (NCT01905150)	Completed/March 2019	G-FLIP/G-FLIP-DM + Vit.C	[26]	ABCG2	[2,16,27]	miR-1246	[16,28]
	In vivo, Phase 1/2 (NCT03410030)	Ongoing/July 2020	Vit.C + Nanoparticle + Paclitaxel + Cisplatin + Gemcitabine	[29]	ALDH1	[2,16,30]	miR-210	[16,31]
	In vivo, Phase 2 (NCT03146962)	Ongoing/December 2021	High dose Vit.C	[32]	CD24	[16]	miR-21	[16,31]
					CD44	[2,16,33]	Let-7	[16,34]
					CD133	[2,16,35]	miR-200 family	[16,34]
					C-Met	[16]	miR-200a	[16,36]
					CXCR4	[16]	miR-143/145 cluster	[16,37]
					Nestin	[16]	miR-145	[16,34]
					Nodal-Activin	[16]	miR-34 family	[16,38]
**Ovarian Cancer**	In vivo, Phase 1/2 (NCT00228319)	Completed/August 2007	Paclitaxel + Carboplatin + Sodium Ascorbate + Vit.C, A & E	[39]	CD24	[16]		
					CD44	[2,16,40]		
					CD177	[16]		
					CD133	[2,16,41]		
**Glioma/Glioblastoma**	In vivo, Phase 2 (NCT02344355)	Ongoing/December 2023	Radiation + temozolomide + Vit.C	[42]	CD15	[16]	miR-145	[22,43]
					CD90	[16]	miR-21	[22,44]
					CD133	[16]	miR-18	[18,45]
					Nestin	[16]	miR-204	[18,46]
					α6-integrin	[16]	miR-128	[18,47]
							miR-23b	[18,48]
**Lung Cancer**	In vivo, Phase 2 (NCT03146962)	Ongoing/December 2021	High dose Vit.C	[32]	ABCG2	[2,16,49]	miR-145	[22,50]
	In vivo, Phase 2 (NCT02420314)	Ongoing/December 2025	Paclitaxel, Carboplatin + Vit.C	[51]	ALDH1	[2,16,52]	miR-191	[18,53]
	In vivo, Phase 2 (NCT02905591)	Ongoing/July 2026	Radiation Therapy + Paclitaxel, Carboplatin + Vit.C	[54]	CD90	[16]	miR-487b	[18,55]
					CD177	[16]		
					CD133	[2,16,56]		
**Colon Cancer**	In vivo, Phase 2 (NCT03146962)	Ongoing/December 2021	Vit.C	[32]	ABCB5	[16]	Let-7	[18,34,57]
					ALDH1	[2,16,58]		
					CD24	[16]		
					CD26	[16]		
					CD29	[16]		
					CD44	[2,16,59]		
					CD133	[2,16,60]		
					CD166	[16]		
					LGR5	[16]		
					β-catenin activity	[16]		
**Leukemia**	In vivo, Phase 2 (NCT03397173)	Ongoing/January 2020	Azacitidine + Vit.C	[61]			miR-27a	[18,62]
	In vivo, Phase 2 (NCT03613727)	Ongoing/October 2022	Vit.C	[63]				
**Lymphoma**	In vivo, Phase 2 (NCT03418038)	Ongoing/March 2024	Salvage Chemotherapy + Vit.C	[64]				
	In vivo, Phase 2 (NCT03613727)	Ongoing/October 2022	Vit.C	[63]				
**Myeloid Leukemia**	In vivo, Phase 2 (NCT03397173)	Ongoing/January 2020	Azacitidine + Vit.C	[61]			miR-130b	[18,65]
	In vivo, Phase 2 (NCT03613727)	Ongoing/October 2022	Vit.C	[63]			miR-29a	[18,66]
							miR-326	[18,67]
							miR-150	[18,68]
**Prostate Cancer**	In vivo, Phase 2 (NCT02516670)	Ongoing/January 2030	Docetaxel + Vit.C	[69]	ALDH1	[2,16,70]	miR-7	[22,71]
					CD44	[2,16,72]	miR-34a	[22,73]
					CD133	[2,16,74]		
					CD166	[16]		
					Trop2	[16]		
					α2β1Integrin	[16]		
					α1Integrin	[16]		
					ABCG2	[2,75]		

## Abbreviations

OH	Hydroxyl radical
α-KGDDs	alpha-ketoglutarate dependent dioxygenases
5-caC	5-carboxycytosine
5-fC	5-formylcytosine
5-hmC	5-hydroxymethylcytosine
5-mC	5-methylcytosine
AKT	Protein kinase B
ALDH	Aldehyde dehydrogenase
BRAF	B-Raf proto-oncogene, serine/threonine kinase
CD	Cluster of differentiation
CSCs	Cancer stem cells
Cu^2+^	Cupric ion
CXCR4	C-X-C chemokine receptor type 4
d-TPP	Dodecyl—tri-phenyl-phosphonium
DHA	Dehydroascorbic acid
DNMT	DNA methyltransferase
EMT	Epithelial-mesenchymal transition
ESCs	Embryonic stem cells
Fe^2+^	Ferrous ion
GAPDH	Glyceraldehyde-phosphate dehydrogenase
GLUT	Glucose transporter
H_2_O_2_	Hydrogen peroxide
HCC	Hepatocellular carcinoma
HIF1α	Hypoxia-inducible factor 1 alpha
IC-50	Inhibitory concentration
IDH	Isocitrate dehydrogenase
iPS	Induced pluripotent stem cells
JAK	Janus kinase
JHDM	Jumonji-C domain-containing histone demethylases
KRAS	KRAS proto-oncogene, GTPase
LGR5	Leucine-rich repeat-containing G-protein coupled receptor 5
miRNA	MicroRNA
mTOR	Mammalian target of rapamycin
NAD^+^	Nicotinamide adenine dinucleotide
NADPH	Nicotinamide adenine dinucleotide phosphate
PARP	Poly (ADP-ribose) polymerase
PBMC	Peripheral blood mononuclear cells
PI3K	Phosphatidylinositol-4,5-bisphosphate 3-kinase
ROS	Reactive oxygen species
SCVT1/2	Sodium dependent Vit.C transporter 1/2
STAT	Signal transducer and activator of transcription
TET	Ten eleven translocation
TPP	Tri-phenyl-phosphonium
Vit.C	Vitamin C
